# Prepared Radix Polygoni Multiflori and emodin alleviate lipid droplet accumulation in nonalcoholic fatty liver disease through MAPK signaling pathway inhibition

**DOI:** 10.18632/aging.205485

**Published:** 2024-01-26

**Authors:** Changyudong Huang, Yiqiong Zhang, Yongjie Xu, Sijia Wei, Tingting Yang, Shuang Wang, Chengcheng Li, Hairong Lin, Xing Li, Shuyun Zhao, Liying Zhu, Wei Pan

**Affiliations:** 1 Guizhou Prenatal Diagnosis Center, Affiliated Hospital of Guizhou Medical University, Guiyang 550004, Guizhou, P.R. China; 2 School of Clinical Laboratory Science, Guizhou Medical University, Guiyang 550004, Guizhou, P.R. China; 3 School of Basic Medical Sciences, Guizhou University of Traditional Chinese Medicine, Guiyang 550004, Guizhou, P.R. China; 4 Reproductive Center, Affiliated Hospital of Guizhou Medical University, Guiyang 550004, Guizhou, P.R. China

**Keywords:** prepared Radix Polygoni Multiflori, emodin, nonalcoholic fatty liver disease, network pharmacology, MAPK signaling pathway

## Abstract

As one of the most common liver diseases, nonalcoholic fatty liver disease (NAFLD) affects almost one-quarter of the world’s population. Although the prevalence of NAFLD is continuously rising, effective medical treatments are still inadequate. Radix Polygoni Multiflori (RPM) is a traditional Chinese herbal medicine. As a processed product of RPM, prepared Radix Polygoni Multiflori (PRPM) has been reported to have antioxidant and anti-inflammatory effects. This study investigated whether PRPM treatment could significantly improve NAFLD. We used recent literature, the Herb database and the SwissADME database to isolate the active compounds of PRPM. The OMIM, DisGeNET and GeneCards databases were used to isolate NAFLD-related target genes, and GO functional enrichment and KEGG pathway enrichment analyses were conducted. Moreover, PRPM treatment in NAFLD model mice was evaluated. The results indicate that the target genes are mainly enriched in the AMPK and *de novo* lipogenesis signaling pathways and that PRPM treatment improves NAFLD disease in model mice. Here, we found the potential benefits of PRPM against NAFLD and demonstrated *in vivo* and *in vitro* that PRPM and its ingredient emodin downregulate phosphorylated P38/P38, phosphorylated ERK1/2 and genes related to *de novo* adipogenesis signaling pathways and reduce lipid droplet accumulation. In conclusion, our findings revealed a novel therapeutic role for PRPM in the treatment of NAFLD and metabolic inflammation.

## INTRODUCTION

Although there is a perceived improvement in quality of life in today’s society, nonalcoholic fatty liver disease (NAFLD), a common metabolic syndrome strongly associated with diabetes, hyperlipidemia, and obesity, affects more than a quarter of the world’s population [1, 2]. However, even though NAFLD seriously threatens public health, there is no FDA-approved treatment for NAFLD [3], and the development of novel drugs requires further study. NAFLD refers to a group of diseases characterized by excessive fat accumulation in the liver, including nonalcoholic steatosis, nonalcoholic steatohepatitis, hepatic fibrosis, hepatic cirrhosis and hepatocellular carcinoma. According to the two-hit hypothesis, which has been widely accepted as the classic pathogenesis of NAFLD [4], hepatic accumulation of lipids acts as the “first hit” and sensitizes the liver to further damage. According to the multiple-hit hypothesis [5], the first hit is still mainly lipid accumulation in hepatocytes caused by insulin resistance, and then the second hit follows: lipid peroxidation damage caused by reactive oxygen species and related events, resulting in steatohepatitis. Steatohepatitis persists, extracellular matrix synthesis is enhanced, and progressive liver fibrosis occurs. Regardless of which hypothesis, abnormal accumulation of lipids in the liver is the initial process before substantial liver damage; thus, treatments decreasing hepatic lipid accumulation could be the focus in coming years. Basically, intrahepatic triglycerides are derived from *de novo* lipogenesis (DNL) and esterification of fatty acids but are consumed via apolipoprotein B assembly, very low-density lipoprotein secretion, intrahepatic lipolysis, and mitochondrial and peroxisome β-oxidation. As one of the pathways involved in triglyceride synthesis, abnormal expression of *de novo* lipogenesis-related factors such as sterol-regulatory element binding proteins (SREBP-1c), ATP citrate lyase (ACLY), acetyl coenzyme A carboxylase 1 (ACC1), and fatty acid synthase (FASN) and dysfunction in lipid metabolic pathways cause lipid deposition and contribute to the development of NAFLD [6]. Thus, exploring treatments that target this pathway requires further studies.

As a traditional Chinese medicine formulation, Radix Polygoni Multiflori (RPM) has been proven to have various beneficial pharmacological effects, including the ability to promote nerve cell growth [7], ameliorate neurasthenia symptoms [8, 9], alleviate oxidative stress damage [10], delay cell aging [11] and improve neurodegenerative disease [12]. Depending on different processing technologies, RPMs are generally divided into two kinds of medicinal materials: raw RPM and prepared RPM (PRPM), although they are from the same plant. PRPM is the product of raw RPM mixed with black bean juice and yellow rice wine, steamed in water and dried [13]. According to the 2020 edition of *the Chinese Pharmacopoeia*, raw RPM has the effects of detoxifying, eliminating carbuncles, intercepting malaria, moistening intestines and relieving defecation, while PRPM has the effects of tonifying the liver, kidney, essence and blood, blacking hair, strengthening muscles and bones, resolving turbidity and regulating lipids. Studies have shown that the main chemical components of raw RPM and PRPM change little, but the content of chemical components varies greatly, which is the main reason for their different efficacies and indications [14]. After processing, compared with raw RPM, the content of stilbene glycosides and anthraquinones in PRPM decreased greatly, but the ratio of anthraquinones to total compounds increased, and most of the bound anthraquinones were converted to free anthraquinones after processing, such as emodin and physcione. Some reports of clinical adverse reactions show that taking RPM can damage the function of the liver and kidney, which is contrary to the theory of traditional Chinese medicine on the function of RPM and contrary to the results of some studies showing that RPM protects the liver and reduces hepatitis and fatty liver. These different outcomes may be related to the dosage of RPM and the content of chemical components of raw RPM and PRPM. It is reported in the literature that *Polygonum multiflorum* has the effect of tonifying the liver and tonifying blood, reducing the anti-inflammation and reducing fat of nonalcoholic fatty liver disease and reducing the incidence of complications of nonalcoholic fatty liver disease, but the mechanism is unknown; and the protective effect of RPM on the liver is contrary to some reports need to be further explained.

Network pharmacology is a method used to conduct multidimensional analyses of drugs, targets and diseases with internet software and omics data [15–17]. It has strong applicability for the prediction of components and their potential therapeutic effects on related diseases in traditional Chinese medicine. In this study, we predicted the related targets of RPMP to protect against nonalcoholic fatty liver disease through network pharmacological analysis and proved that emodin is the main component of *Polygonum multiflorum* that protects against nonalcoholic fatty liver disease at the cellular and animal levels.

## MATERIALS AND METHODS

### Network pharmacology analysis

By conducting literature searches in coming years [18–22] and searching in the high-throughput experiments of traditional Chinese medicine and the reference database HERB (http://herb.ac.cn/) using the keyword He Shou Wu, the drug components of PRPM were obtained. The SDF files and 2D structures of the drug components were searched and collected through the PubChem database (http://pubchem.ncbi.nlm.nih.gov). In Swiss ADME (http://www.swissadme.ch/) software, using the SDF file of the drug components as an index, we obtained relevant information about the drug components. The compounds were then screened, and the screening criteria included oral bioavailability (OB) ≥0%, high gastrointestinal absorption (Gl absorption) score, and three out of five Druglikeness parameters (Lipinski, Ghose, Veber, Egan, and Muegge) set to “Yes”. In the Swiss Target Prediction software (http://www.swisstargetprediction.ch/), the selected compounds of He Shou Wu were used as keywords for retrieval to obtain the potential target gene names of the components. Gene names were validated using the UniProt (http://www.uniprot.org/) database.

A total of 430 targets of PRPM were identified using SwissTargetPrediction (http://www.swisstargetprediction.ch/), and 479 potential targets were collected from the GeneCards (https://www.genecards.org/), DisGeNET (https://www.disgenet.org/), DrugBank (https://go.drugbank.com/) and OMIM databases (https://www.omim.org/). A total of 479 target symbols associated with NAFLD were identified using the OMIM database. The enrichment of RPM potential targets and NAFLD-related targets in the GO pathway and KEGG pathway was subsequently analyzed using DAVID (https://david.ncifcrf.gov/), and a network map including components, target gene symbols, KEGG enriched pathways, and related diseases was constructed in Cytoscape 3.9.1.

### Microarray data and screening differentially expressed genes

Two gene expression profiles, GSE89632 and GSE96971, were obtained from the National Center for Biotechnology Information Gene Expression Omnibus (GEO) database (http://www.ncbi.nlm.nih.gov/geo). Differentially expressed genes between NAFLD and normal tissue samples were screened via GEO2R (http://www.ncbi.nlm.nih.gov/geo/geo2r/). The established Bioconductor R package was used to analyze the GEO data. Criteria were set at *P*-values (adj. *P*) < 0.05 and |logFC| > 1 to select common DEGs from the four datasets for further analysis. In addition, enrichment analyses of GO pathways and KEGG pathways were performed in DAVID (https://david.ncifcrf.gov/).

### Preparation of prepared Radix Polygoni Multiflori

The method of PRPM treatment was based on our group’s previous research [7, 12]. A total of 500 g of raw RPM (Tongji Tang Pharmacy, Guizhou) was steeped in water for 1 h, heated and boiled for 60 min, and the liquid was removed; the decoction was repeated twice by continuing to add water, and the liquid was combined 3 times and passed through gauze to remove the residue. Forty-eight hours later, the liquid was evaporated under reduced pressure at 100°C and stored in an evaporator. The Chinese Pharmacopoeia records that the dose of PRPM for rats is 0.63–1.26 g/kg, so the dosage for the low-dose group is 1 g/kg/d and 2 g/kg/d for the high-dose group.

### Experimental animals

Thirty db/db mice and 10 db/m mice aged between 6 and 8 weeks were obtained from Junke Biological Co., Ltd., (Nanjing, China). Db/m mice were regarded as the normal control (NC) group. Db/db mice were randomly assigned to 3 groups: the NAFLD control group, low PRPM dose (1 g/kg/d) group (low PRPM), and high PRPM dose (2 g/kg/d) group (high PRPM). Mice in each group were housed at the Animal Centre of GuiZhou Medical University in an environment with a 12 h light/dark cycle in a temperature range of 23–25°C (relative humidity: ~50%). The mice received standard laboratory chow pellets, and water was supplied ad libitum. After 6 weeks of adaptive feeding, the NC group and NAFLD group received saline gavage, while the low PRPM and high PRPM groups received PRPM gavage at the corresponding dose for 8 weeks. After 8 weeks of intervention, the mice were anesthetized by intraperitoneal injection of sodium pentobarbital (100 mg/kg). Then, all mice were sacrificed by cervical dislocation. The experiments were conducted in strict accordance with the Principles of Laboratory Animal Care (People’s Republic of China), and the experiments were approved by the Animal Care and Use Committee of Guizhou Medical University (approval no. 2001342; Guiyang, China).

### TG, ALT, and ELISA

Manufactured kits (Nanjing Jiancheng Institute of Biological Engineering) were used for the determination of mouse serum TG (A110-2-1) and ALT (C009-3-1), and ELISA kits (Shanghai ELISA Biotechnology Co., Ltd.) were used for the determination of mouse serum insulin, IL-6 (ml063159) and TNF-α (ml002095). Fifty microliters of mouse serum diluted 1:1 with assay diluent was added to the reaction wells. Fifty microliters of the diluted standards and 50 μl of the test samples were added to the reaction wells. The reaction plate was covered, gently shaken and mixed, and incubated at 37°C for 1 hour. The plate was washed 3 times with washing solution. Eighty microliters of high-affinity streptavidin-HRP solution was added to each well, shaken gently and incubated at 37°C for 30 minutes. After 3 washes, 50 μl each of a mixed substrate A and B solution was added to each well, shaken gently, mixed well, and incubated at 37°C for 10 minutes in the dark. Fifty microliters of termination solution was quickly added, and the OD of each well was measured at 450 nm.

### Hematoxylin and eosin (H&E) staining

After the mice were fasted for 12 h and anesthetized by intraperitoneal injection of sodium pentobarbital (100 mg/kg), they were intracardially perfused using 0.9% sodium chloride solution and sacrificed by cervical dislocation. Liver tissues were isolated and stored in formalin and liquid nitrogen. After paraffin embedding, liver sections were deparaffinized with xylene and rehydrated. For H&E staining, liver sections were processed according to standard procedures, and the morphological changes were observed under a microscope (Nikon Eclipse C1).

### Immunohistochemistry

For immunohistochemical (IHC) staining, for antigen retrieval, rehydrated sections were immersed in citrate buffer (pH 6.0) at 121°C for 2 minutes and then slowly cooled to room temperature. Endogenous peroxidase activity was blocked with 3% hydrogen peroxide (H_2_O_2_) for 25 minutes in the dark. Nonspecific binding was blocked with 3% bovine serum albumin for 30 minutes. After covering with the primary antibody (SREBP1 1:100, Cat No. 14088-1-AP, Proteintech, Wuhan, China), (P38 1:250, Cat No. 14064-1-AP, Proteintech, Wuhan, China), (P-P38 1:250, Cat No. 28796-1-AP, Proteintech, Wuhan, China), (ERK1/2 1:250, Cat No. 11257-1-AP, Proteintech, Wuhan, China), (P-ERK1/2 1:100, Cat No. 28733-1-AP, Proteintech, Wuhan, China) overnight at 4°C in a humidified chamber, the sections were reacted with HRP-conjugated goat anti-rabbit antibody (1:500; Proteintech, Ltd, Wuhan, China) for 30 min at 37°C, followed by detection with 3,3′-diaminobenzidine (DAB) (Dako, Glostrup, Denmark) and counterstaining with Mayer’s hematoxylin solution (Sigma-Aldrich, St. Louis, MO, USA), and changes were observed under the microscope. The histoscore, also known as the H-score, was used for semiquantitative evaluation of immunohistochemistry. The H-score accounts for both the percentage and intensity of positive staining. It is calculated by multiplying the percentage of mildly stained cells by 1, the percentage of moderately stained cells by 2, and the percentage of strongly stained cells by 3.

### Cell lines and coculture assay

The HepG2 cell line, which was derived from a hepatocellular carcinoma cell line, was a gift from our group by Professor Zhu. For cell recovery, cells frozen in liquid nitrogen were rapidly transferred to a 37°C incubator for complete thawing and then centrifuged at 800 rpm for 5 min in a 15 ml centrifuge tube filled with culture medium. The supernatant was discarded, and the cells were resuspended in T-25 culture flasks with an appropriate amount of culture medium. After the cells in the culture flask had grown to 80–90% confluence, 2 ml of trypsin was added to digest the cells for 3 min, 4 ml of culture medium was added to terminate the digestion, and then the cells were added to the tube and centrifuged for 5 min at 800 rpm. Then, the cells were resuspended in a new T-25 culture flask. The cells were treated with a specific concentration of emodin (Beijing Solab Technology Co., Ltd., China) when they were in their logarithmic growth phase, and the proteins were extracted after 24 h. The culture conditions were all in a mixture of 10% FBS (Gibco Life Technologies, Waltham, MA, USA) and 1% penicillin-streptomycin (Invitrogen Life Technologies, Waltham, MA, USA) in an incubator at 37°C, 5% CO_2_ and 95% relative humidity. A total of 4 × 105 cells per well were inoculated in 6-well plates and 6-well plates containing cell climbing slices. Cells were incubated with 100 μM oleic acid (O8291, Solarbio, China) and 200 μM palmitic acid (H8780, Solarbio, China) for lipid accumulation models. After growing to 80%, medium containing different concentrations of emodin (E8390, Solarbio, China) was added for 24 hours, and total cell protein or cell climbing slices were extracted. To investigate the correlation between the efficacy of PRPM and emodin with the MAPK signaling pathway, HepG2 cells were treated with 15 nM MEK inhibitor selumatinib (), 5 μM p38 inhibitor losmapimod, and 10 nM MAPK activator diprovocim for 24 hours.

### Immunofluorescence staining

Immunofluorescence staining and microscopy were performed as described previously [23], and the concentrations of antibodies were as follows: SREBP1 1:100 (Cat No. 14088-1-AP, Proteintech, Wuhan, China), P38 1:250 (Cat No. 14064-1-AP, Proteintech, Wuhan, China), P-P38 1:250 (Cat No. 28796-1-AP, Proteintech, Wuhan, China), ERK1/2 1:250 (Cat No. 11257-1-AP, Proteintech, Wuhan, China), and P-ERK1/2 1:100 (Cat No. 28733-1-AP, Proteintech, Wuhan, China). To perform semiquantitative analysis, the fluorescence intensity of immunofluorescence was measured via ImageJ.

### Oil red O staining and nil red staining

Oil red O staining (D027-1-1, Nanjing Jiancheng Institute of Biological Engineering, China) and Nile red staining (N8440, Solarbio, Beijing, China) were applied to evaluate lipid accumulation in mouse livers and HepG2 cells with different treatments. After sacrifice, sections (9 μm thick) were cut from the frozen mouse liver tissues and stained with freshly diluted Oil Red O staining solution for 20 min. After different treatments for 24 h, the cell medium was removed. For Oil red O staining, cells were treated with 4% paraformaldehyde for 15 minutes and washed with double-distilled water and 60% isopropanol for 10 seconds. Oil Red O staining solution was mixed with diluent at a ratio of 5:2 and passed through a 0.22 μm filter, and the sections were stained for 30 minutes protected from light and then washed with double-distilled water. The sections were restained with hematoxylin for 5 minutes and then washed with double-distilled water. Then, the tissue sections were sealed with an aqueous sealer. For Nile red O staining, cell sections were fixed with 10% paraformaldehyde for 10 min at room temperature. Then, the sections were incubated with 2 μg/mL Nile red for 15 min. Images were acquired by fluorescence microscopy (Nikon Eclipse C1). The semiquantification of Oil Red O staining was conducted by measuring the area of staining above a predefined constant area. This measurement was performed using ImageJ. To perform semiquantitative analysis, the fluorescence intensity of Nile red staining was measured using ImageJ software.

### Western blot analysis

High-efficiency RIPA tissue/cell lysate (Beijing Soleibo Technology Co., Ltd., China) (containing PMSF) was used to lyse liver cancer tissue or cells to extract proteins, and then they were separated on a 10% or 12% gel with SDS-polyacrylamide. Samples (20 μg) were wet-transferred into 0.22 μm PVDF membranes (Millipore, Burlington, MA, USA) and covered with 5% nonfat milk for 2 hours at room temperature, followed by incubation with primary antibodies overnight (SREBP1 1:1000, Cat No. 14088-1-AP, Proteintech, Wuhan, China), (ACC1 1:1000, Cat No. 21923-1-AP, Proteintech, Wuhan, China), (FASN 1:1000, Cat No. 10624-2-AP, Proteintech, Wuhan, China), (ACLY 1:2000, Cat No. 15421-1-AP, Proteintech, Wuhan, China), (P38 1:1000, Cat No. 14064-1-AP, Proteintech, Wuhan, China), (P-P38 1:1000, Cat No. 28796-1-AP, Proteintech, Wuhan, China), (ERK1/2 1:1000, Cat No. 11257-1-AP, Proteintech, Wuhan, China), (P-ERK1/2 1:1000, Cat No. 28733-1-AP, Proteintech, Wuhan, China), (GAPDH 1:10000, Catalog No. 10494). The membranes were washed 3 times in TBS with 0.1% Tween-20 buffer for 10 min each, reacted with secondary antibody for 2 h at room temperature, and then washed 3 times in TBST buffer for 10 min each minute. Bands were visualized with enhanced chemiluminescence Western blot detection reagents (Advansta, Inc., Menlo Park, CA, USA), and relative protein levels were quantified by using ImageJ.

### RT-qPCR

RT-qPCR was performed to analyze the mRNA levels of SREBP-1c, ACC1, FASN, and ACLY. Total RNA was extracted from mouse liver tissue as well as HepG2 cells using TRIzol reagent (Life Technologies, Thermo Fisher Scientific), and mRNA was then reverse transcribed into cDNA via a reverse transcription kit (DRR036A, TaKaRa Biotechnology Co., Ltd., Japan). The cDNA was then amplified according to the instructions of the SYBR fluorescence quantification kit (DRR420A, TaKaRa Biotechnology Co., Ltd., Japan). The primers were designed in NCBI Primer-BLAST (https://blast.ncbi.nlm.nih.gov/Blast.cgi), and the specific primer sequences are given as listed below. Fold changes in gene expression were calculated by the 2^−ΔΔCT^ method using GAPDH as a control.

Primers for RT-qPCR for human genes were as follows: GAPDH-F: ACCCAGAAGACTGTGGATGG; GAPDH-R: CACATTGGGGGTAGGAACAC; SREBP-1-F: ACTTCTGGAGGCATCGCAAGCA; SREBP-1-R: AGGTTCCAGAGGAGGCTACAAG; FASN-F: TTCTACGGCTCCACGCTCTTCC; FASN-R: GAAGAGTCTTCGTCAGCCAGGA; ACC1-F: TTCACTCCACCTTGTCAGCGGA; ACC1-R: GTCAGAGAAGCAGCCCATCACT; ACLY-F: GCTCTGCCTATGACAGCACCAT; and ACLY-R: GTCCGATGATGGTCACTCCCTT. Primers for RT-qPCR for mouse genes were as follows: GAPDH-F: CATCACTGCCACCCAGAAGACTG; GAPDH-R: ATGCCAGTGAGCTTCCCGTTCAG; SREBP-1-F: CGACTACATCCGCTTCTTGCAG; SREBP-1-R: CCTCCATAGACACATCTGTGCC; FASN-F: CACAGTGCTCAAAGGACATGCC; FASN-R: CACCAGGTGTAGTGCCTTCCTC; ACC1-F: GTTCTGTTGGACAACGCCTTCAC; ACC1-R: GGAGTCACAGAAGCAGCCCATT; ACLY-F: AGGAAGTGCCACCTCCAACAGT; and ACLY-R: CGCTCATCACAGATGCTGGTCA.

### Statistical analysis

Statistical analyses were performed with SPSS 17.0 software (SPSS, Chicago, IL, USA). Data were analyzed using two independent sample *t*-tests. Each experiment was performed three times. *P* < 0.05 was considered to indicate a statistically significant difference.

### Availability of data and materials

The data that support the findings of this study are available from the corresponding author upon reasonable request.

## RESULTS

### Network pharmacology analysis of prepared Radix Polygoni Multiflori for the treatment of NAFLD

**Figure 1:**
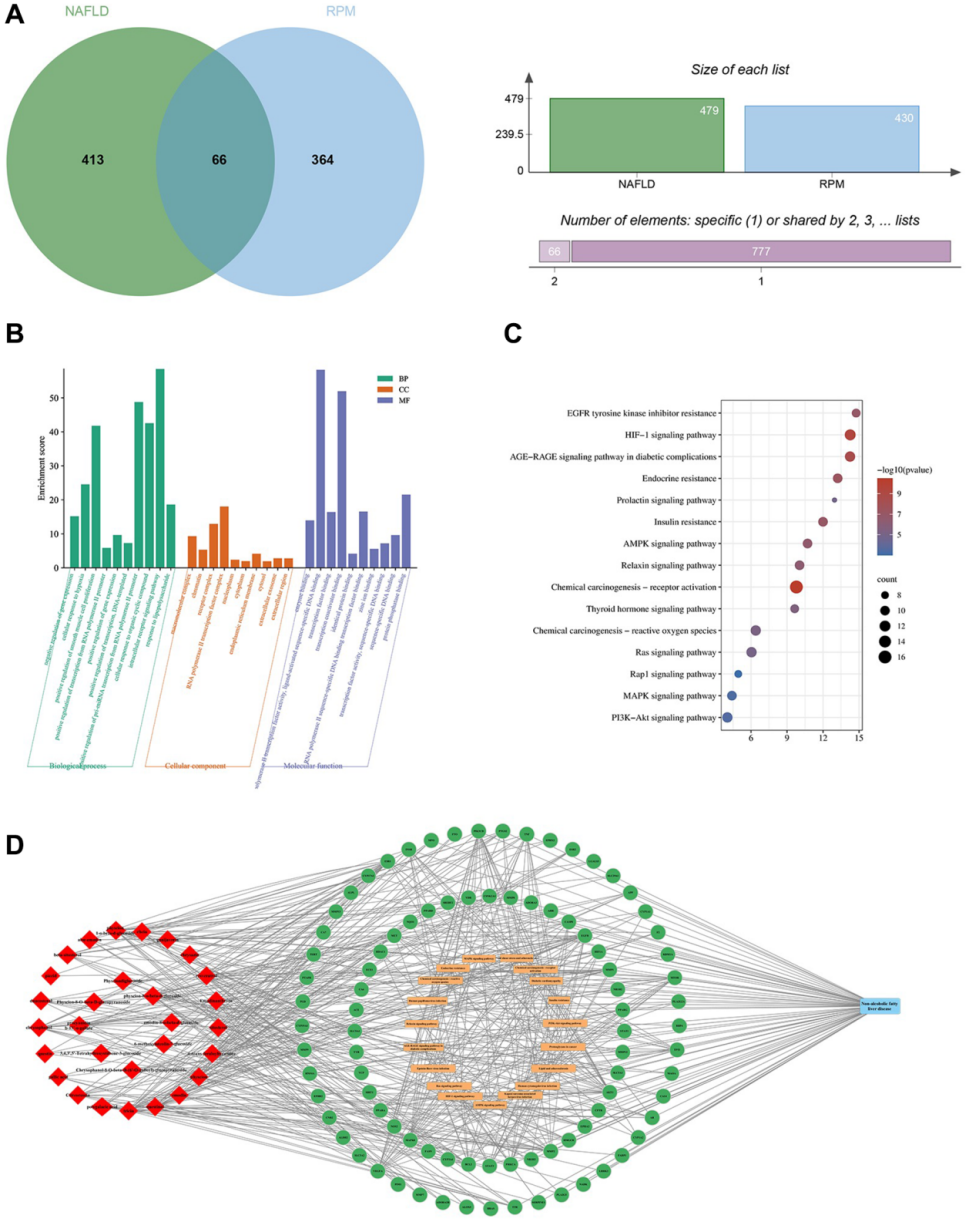
**Enrichment analysis of the anti-NAFLD mechanisms of PRPM.** (**A**) Sixty-six common targets of NAFLD and PRPM. (**B**) The top 10 significantly enriched (*p* < 0.05) terms in BP, CC and MF of GO analysis. The Y-axis represents the enrichment count of the target, and the X-axis represents the GO category of the target gene. (**C**) The top 15 pathways with significant enrichment (*p* < 0.05) were selected. The Y-axis represents the pathway, while the colors represent the significance of differential enrichment, and the size of the circles represents the number of genes. (**D**) Ingredient-target-pathway network of PRPM. The red rhombuses indicate ingredients, the green circles indicate targets, and the orange rounded rectangle indicates pathways.

After screening and filtering in the HERB database, 29 candidate ingredients of PRPM, including anthraquinone-related compounds and adipoids, were selected based on oral bioavailability (OB) greater than or equal to 30% or drug-like properties (DL) greater than or equal to 0.18 (considering that numerous studies have demonstrated the beneficial effects of the ingredient resveratrol on fatty liver, it was also treated as one of the candidate ingredients, even though it did not meet the screening criteria). The OB and DL scores of each candidate are shown in Supplementary Table 1. After using SwissTargetPrediction software, 430 potential targets of PRPM were identified. Moreover, in total, 479 targets associated with NAFLD were collected from databases including the GeneCards, DisGeNET, DrugBank and OMIM databases. Sixty-six gene symbols between the RPM potential targets and the NAFLD-associated targets overlapped, as shown in Figure 1A, and Gene Ontology (GO) and Kyoto Encyclopedia of Genes and Genomes (KEGG) enrichment analyses were performed in DAVID (Figure 1B, 1C). Finally, a network map among the compound, target genes, KEGG pathways, and diseases was drawn in Cytoscape 3.9.1 (Figure 1D). Network pharmacology analysis of PRPM revealed that these 66 target genes were associated with biological process pathways such as cellular response to hypoxia and oxidative stress and regulation of apoptotic processes. The genes were enriched in cellular components such as macromolecular complex, chromatin, receptor complex, and RNA polymerase II transcription factor complex and in molecular function pathways such as transcription factor binding, enzyme binding, transcription coactivator binding, RNA polymerase II transcription factor activity, ligand-activated sequence-specific DNA binding, and identical protein binding. KEGG pathway analysis revealed that the genes were enriched in the HIF-1 signaling pathway, AMPK signaling pathway, AGE-RAGE signaling pathway associated with diabetic complications, insulin resistance, and MAPK signaling pathway. As an important protein kinase, AMPK (adenosine 5′-monophosphate (AMP)-activated protein kinase) is widely distributed in tissues with high energy metabolism, such as liver, fat and skeletal muscle, and is known as the “cellular energy homeostasis receptor”. This pathway has become a major target for the prevention and treatment of NAFLD. Additionally, the HIF-1 signaling pathway and AGE-RAGE signaling pathway have been reported to be potential targets for NAFLD treatment. After network pharmacology analysis, we found that some ingredients of PRPM might target and protect against hepatic lipid accumulation and inflammation and be potential therapies for NAFLD.

**Figure 2:**
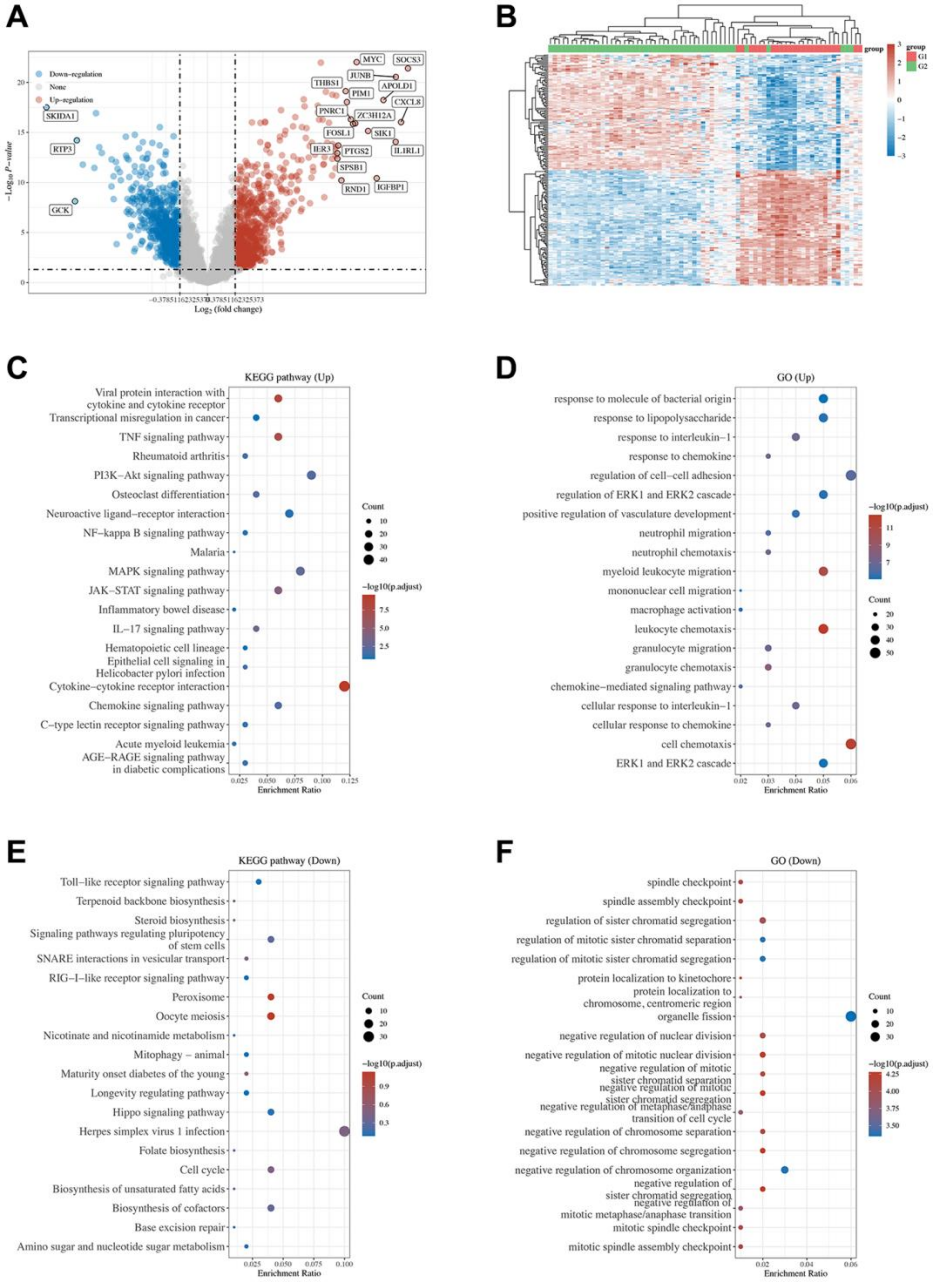
**Volcano plot and heatmap of differentially expressed genes and the functional enrichment of KEGG and GO.** (**A**) Volcano plot was constructed using the fold change values and P-adjust; red indicates upregulation, while blue indicates downregulation. (**B**) Heatmap of the differential gene expression; different colors represent the trend of gene expression in different tissues. The top 50 upregulated genes and the top 50 downregulated genes are shown in this figure. (**C**–**F**) The enriched KEGG signaling pathways and GO functions were selected to demonstrate the primary biological actions of major potential mRNAs. The abscissa indicates the gene ratio, and the enriched pathways are presented in the ordinate. Colors indicate the significance of differential enrichment, and the size of the circles indicates the number of genes. In the enrichment results, *p* < 0.05 and FDR <0.05 were considered meaningful pathways (enrichment score with −log10 (P) of more than 1.3).

Considering that NAFLD has a complex pathogenesis, multiple factors and pathways can accelerate the progression of NAFLD. To explore which pathways varied the most or might promote the development of NAFLD, two microarray datasets from GEO (GSE89632 and GSE96971) were downloaded, and the gene expression profiles of hepatic tissues from 24 control and 48 NAFLD patients were gathered (Figure 2). After GO and KEGG analyses, we found that the mitogen-activated protein kinase (MAPK) signaling pathway and its members extracellular regulated protein kinases 1/2 (ERK1/2) were enriched in the upregulated genes. Studies have proven that the MAPK signaling pathway plays important roles in hepatocellular carcinoma, the later stages of NAFLD after steatosis in the liver, hepatitis and hepatic cirrhosis [24]. Meanwhile, disruption of p38α promotes steatohepatitis and is involved in macrophage polarization [25]. Therapies targeting MAPK have also shown positive effects on NAFLD, suggesting that MAPK might play an important role in NAFLD [26–28]. Furthermore, studies have also found that AMPK signaling is directly regulated by MAPK and constrains the AMPK-driven oxidative phosphorylation of biomaterials [29].

### Prepared Radix Polygoni Multiflori improves liver function in mice with nonalcoholic fatty liver disease

**Figure 3:**
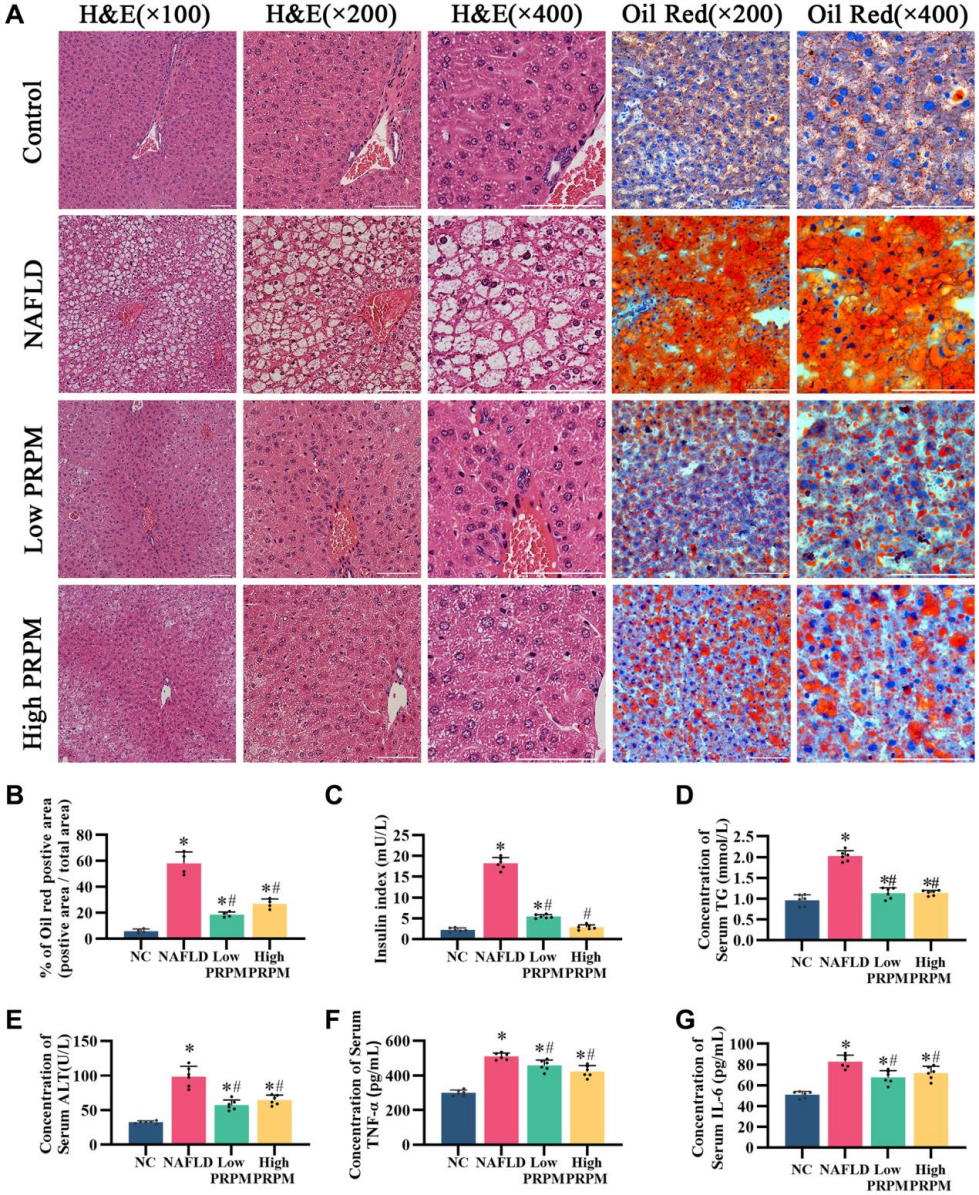
**PRPM improves nonalcoholic fatty liver disease.** (**A**) H&E staining, ×100, ×200, ×400; Oil red staining, ×200, ×400. (**B**) Quantitative analysis of the Oil red O staining-positive area (%). (**C**–**G**) ELISA analysis of the insulin index, serum triglycerides (TG), alanine aminotransferase (ALT), tumor necrosis factor-α (TNF-α), and interleukin-6 (IL-6). ^*^indicates a comparison with NC, *P* < 0.05. ^#^indicates a comparison with NAFLD, *P* < 0.05. The white line indicates 50 μm.

According to the literature, db/db mice are used to construct an NAFLD animal model. Therefore, we successfully constructed a NAFLD model using db/db mice and administered PRPM treatment. The components of emodin, tetrahydroxystilbene glucoside, and physcion were detected via UPLC-MS/MS (Supplementary Materials and Method, Supplementary Figure 1, Supplementary Table 2). After 8 w of PRPM intervention, we assessed the morphology of the liver tissue using hematoxylin and eosin (H&E) staining and discovered that PRPM decreased the formation of hepatic fat vacuoles and reduced fat accumulation (Figure 3A). Later, we tested mice for insulin resistance and examined total triglycerides, serum alanine transaminase, serum TNF-α, and serum IL6 (Figure 3C–3G), which are related to liver function and inflammation status in mice, and found that insulin function and liver function improved in the PRPM intervention group, while they were abnormal in the nonintervention group.

### PRPM and emodin improved MAPK gene disruption in NAFLD

Studies have proven that the MAPK signaling pathway plays important roles in hepatocellular carcinoma, the later stages of NAFLD after steatosis in the liver, hepatitis and hepatic cirrhosis [24]. Meanwhile, disruption in p38α promotes steatohepatitis and is involved in macrophage polarization [25]. Therapies targeting MAPK have also shown positive effects on NAFLD, suggesting that MAPK might play an important role in NAFLD [26–28].

**Figure 4:**
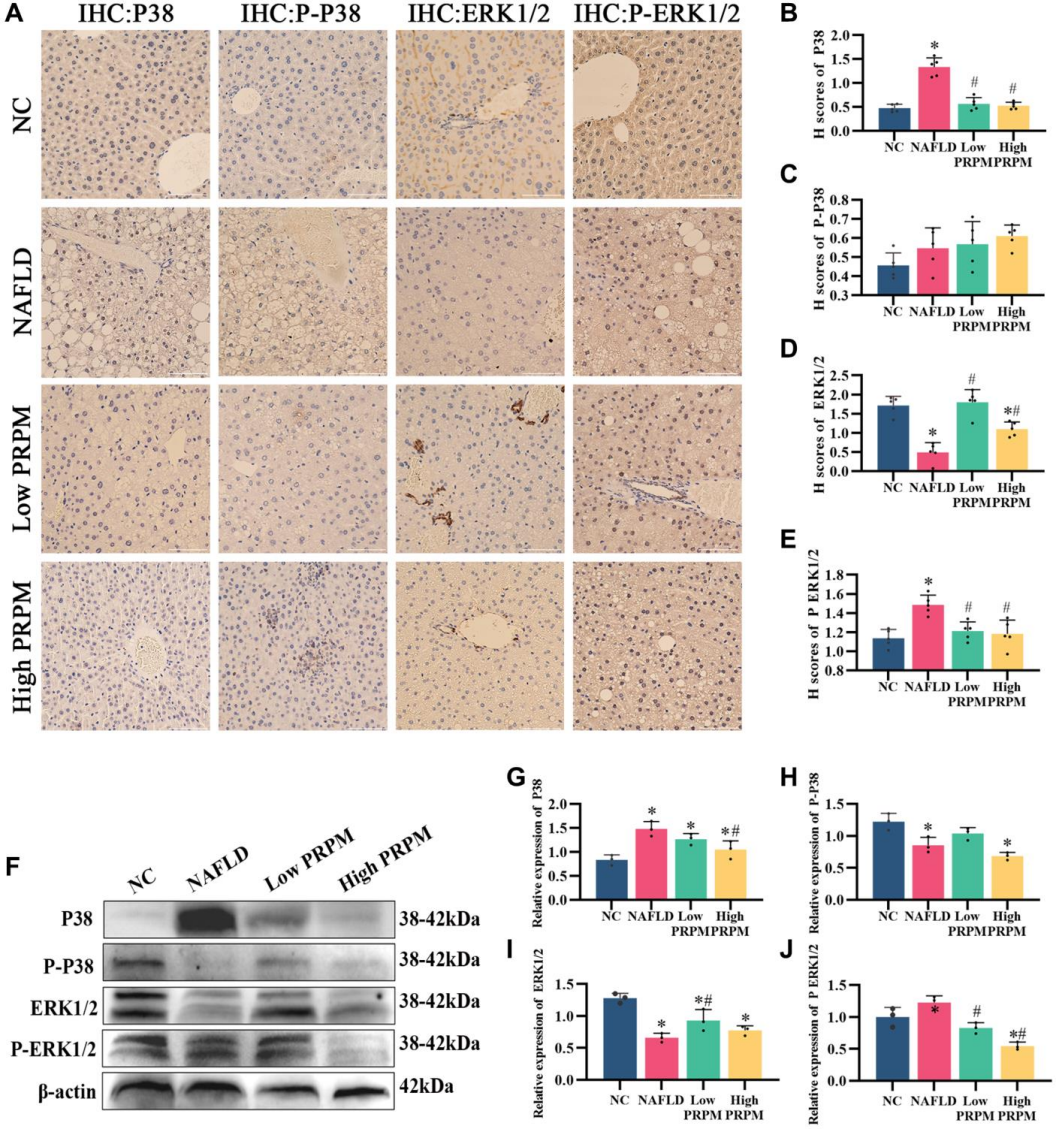
**PRPM mediates the MAPK signaling pathway in NAFLD.** (**A**) IHC staining of P38, phosphorylated P38, ERK1/2 and phosphorylated ERK1/2 in NAFLD mouse models, ×200, ×400. (**B**–**E**) Correlation analysis of H-scores of P38, phosphorylated P38, ERK1/2 and phosphorylated ERK1/2. (**F**) Representative Western blot of P38, phosphorylated P38, ERK1/2 and phosphorylated ERK1/2. (**G**–**J**) Western blot analysis. ^*^indicates comparison with NC, *P* < 0.05. ^#^indicates a comparison with NAFLD, *P* < 0.05. The white line indicates 50 μm.

**Figure 5:**
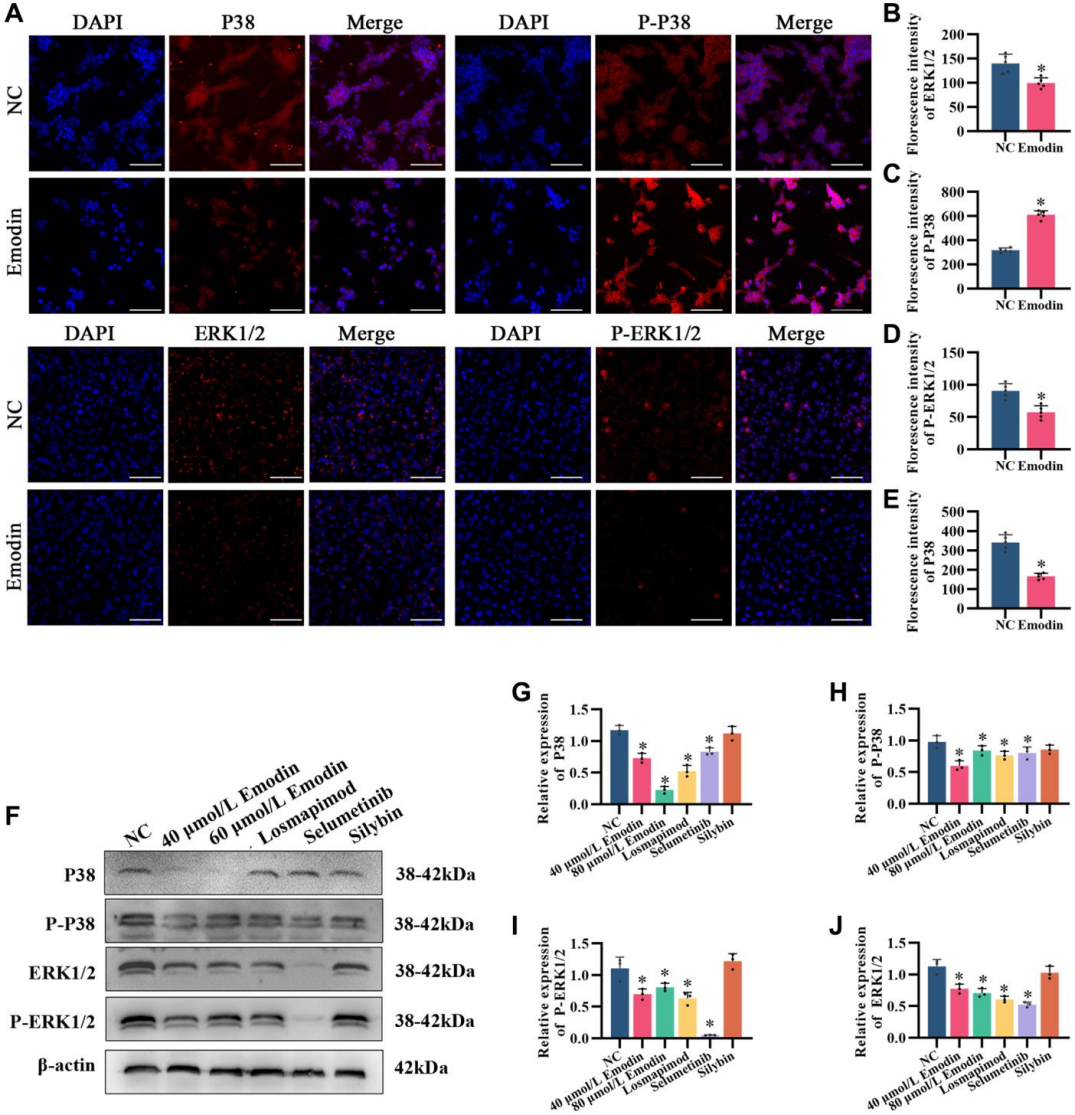
**Emodin mediates the MAPK signaling pathway in HepG2 cells.** (**A**) IF staining of P38, phosphorylated P38, ERK1/2 and phosphorylated ERK1/2 (200×) in HepG2 cells. (**B**–**E**) Florescence intensity analysis of P38, phosphorylated P38, ERK1/2 and phosphorylated ERK1/2. (**F**) Representative Western blot of P38, phosphorylated P38, ERK1/2 and phosphorylated ERK1/2. (**G**–**J**) Western blot analysis. ^*^indicates a comparison with NC, *P* < 0.05. The white line indicates 50 μm.

To demonstrate whether PRPM and its active ingredient emodin could inhibit the activation of MAPK and reduce its associated protein in the liver, we collected mouse liver tissues for immunohistochemical detection (Figure 4A–4E). The levels of the MAPK pathway-related proteins P38 and ERK1/2 and their phosphorylation changed significantly in NAFLD mouse liver tissue, while the alterations were partially recovered after PRPM intervention. According to the bioinformatics results and studies [24, 27, 30], differentially expressed genes were enriched in the MAPK signaling pathway, while the ERK1 and ERK2 cascades were upregulated in the NAFLD group. We verified this finding using Western blotting, and the findings were consistent with the immunohistochemical results (Figure 4F–4J). Compared to the NAFLD groups, the expression levels of p38 increased, while its phosphorylation decreased. In contrast, the expression levels of ERK1/2 decreased, while its phosphorylation increased (Figure 4F–4J). To support the results of this experiment, we conducted an *in vitro* test to detect MAPK-related indicators in cells using immunofluorescence (Figure 5A–5E). The results showed that P38 and ERK1/2 expression decreased after emodin treatment using emodin, and phosphorylated P38 and phosphorylated ERK1/2 expression increased; these results were also verified by Western blotting (Figure 5F–5J). These findings were slightly different than what we found in the mice. Although the expression levels of p38 increased while its phosphorylation decreased, the expression levels of ERK1/2 did not increase, which showed the opposite results in the cell models. Meanwhile, as one of the most common health products for NAFLD treatments, silybin, the main ingredient of milk thistle [31–33], did not show significant effects on the MAPK signaling pathway.

### PRPM and emodin mediate the AMPK signaling pathway in NAFLD

**Figure 6:**
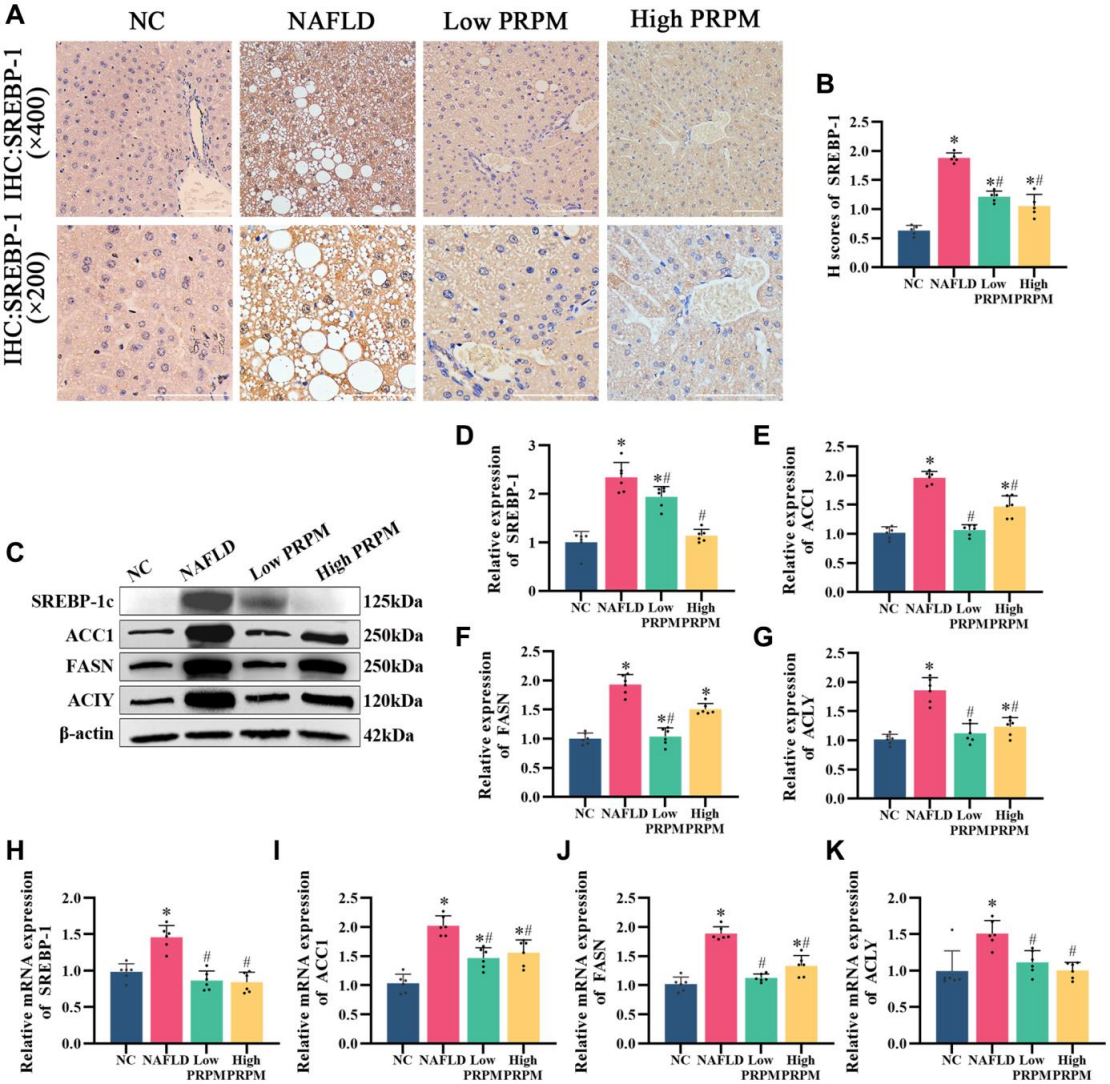
**PRPM mediates the AMPK signaling pathway in NAFLD.** (**A**) IHC staining of SREBP-1, ×200, ×400. (**B**) A correlation analysis of the H-scores of SREBP-1. (**C**) Representative Western blot of SREBP-1, ACC1, FASN, and ACLY. (**D**–**G**) Western blot analysis. (**H**–**K**) Relative mRNA levels of SREBP-1, ACC1, FASN, and ACLY (ratio: measured mRNA/GAPDH mRNA). ^*^indicates comparison with NC, *P* < 0.05. ^#^indicates a comparison with NAFLD, *P* < 0.05. The white line indicates 50 μm.

Abnormal excessive lipid accumulation is the main cardiovascular manifestation of NAFLD, and disturbance of the *de novo* lipogenesis pathway strongly participates in this process. In the network pharmacology analysis, we found that PRPM treatment for NAFLD was related to the AMPK signaling pathway. Here, we found that after PRPM treatment, lipogenesis-related proteins such as ACC1, FASN, and ACLY were mainly regulated by SREBP1, the downstream factor of the AMPK signaling pathway. A previous study by Yu et al. showed that PRPM downregulated AMPK/SREBP-1 in a NAFLD zebrafish model [34], so we also explored the actions of PRPM on the *de novo* lipogenesis pathway (DNL) (Figure 6). Immunohistochemistry (IHC) of SREBP-1c showed that as the key factor of DNL, SREBP-1c was highly expressed in db/db mice compared with db/m mice (Figure 6A, 6B). After PRPM treatment, the expression level of SREBP-1c decreased, and Western blot and q-PCR results (Figure 6C–6K) showed the same results, which were consistent with Yu et al.’s study. Moreover, the expression levels of ACC1, FASN, and ACLY also simultaneously decreased (Figure 6C–6K). In conclusion, our results suggest that PRPM treatment can improve NAFLD by inhibiting the expression of *de novo* lipogenesis-related proteins, and the above results indicate that PRPM treatment can dramatically improve lipid accumulation in NAFLD model mice.

**Figure 7:**
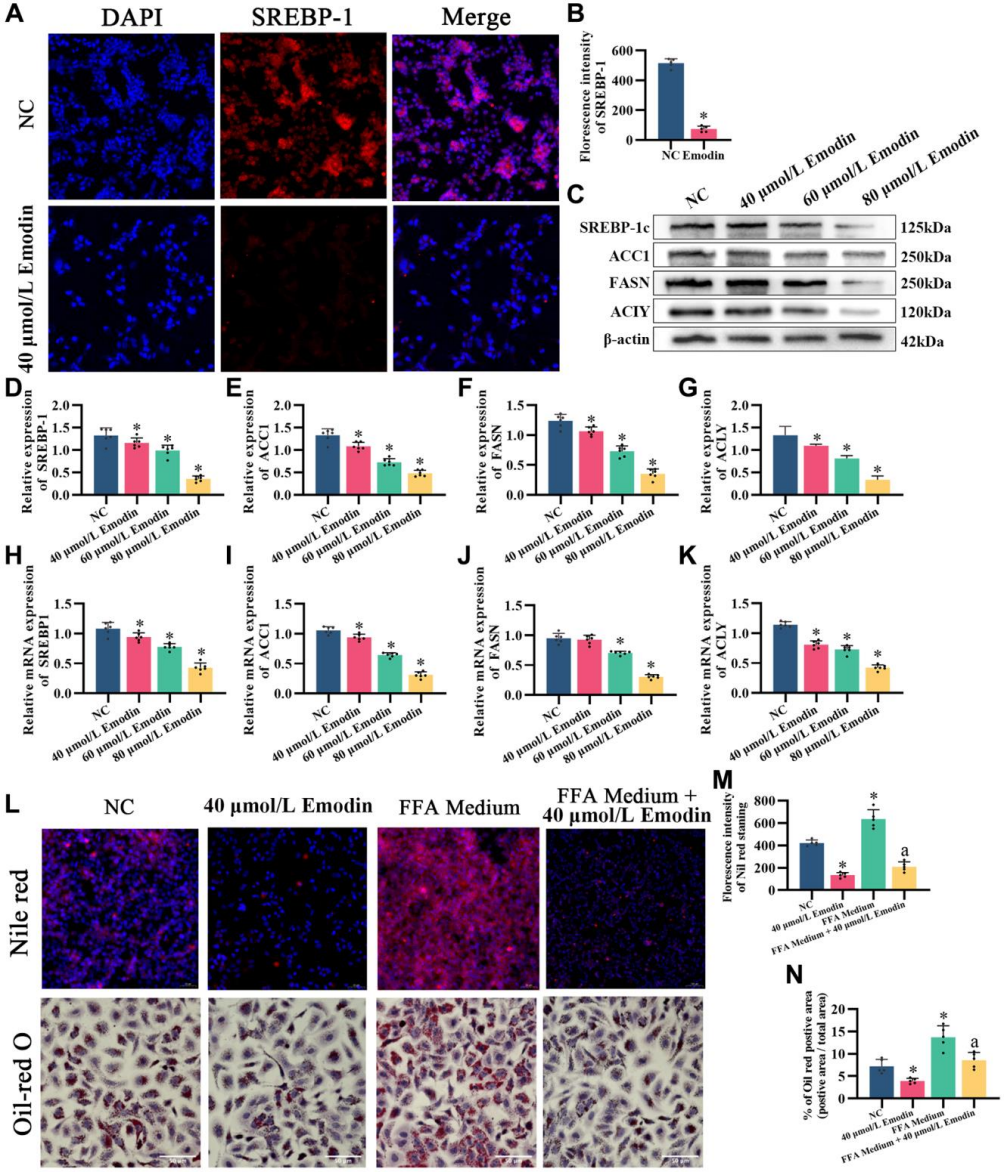
**Emodin mediates the AMPK signaling pathway in HepG2 cells.** (**A**) IF staining of SREBP-1. (**B**) Florescence intensity analysis of SREBP-1, ×200. (**C**) Representative Western blot of SREBP-1, ACC1, FASN, and ACLY. (**D**–**G**) Western blot analysis. (**H**–**K**) Relative mRNA levels of SREBP-1, ACC1, FASN, and ACLY (ratio: measured mRNA/GAPDH mRNA). (**L**–**N**) Nile red and Oil red O staining and analysis. ^*^indicates comparison with NC, *P* < 0.05. A indicates a comparison with FFA Medium, *P* < 0.05. The white line indicates 50 μm.

Later, whether emodin also downregulates the *de novo* lipogenesis pathway was tested. Immunofluorescence of SREBP-1c showed that compared with the normal control, emodin downregulated SREBP-1c (Figure 7A, 7B). Lipid accumulation cell models were established via combined oleic acid and palmitic acid (free fatty acid medium, FFA medium) treatments. Nile red and Oil red O staining showed that emodin intervention reduced lipid droplets in cells caused by excessive fatty acid production (Figure 7L–7N), which occurred when the SREBP-1c expression level was diminished. Western blot and q-PCR results also showed that emodin downregulated the intracellular protein expression levels of SREBP-1c, ACC1, FASN, and ACLY (Figure 7C–7K), which was in accordance with the effects of PRPM treatment in the livers of the NAFLD model mice.

### The MAPK activator diprovocim restored the inhibitory effect of emodin on the AMPK signaling pathway

**Figure 8:**
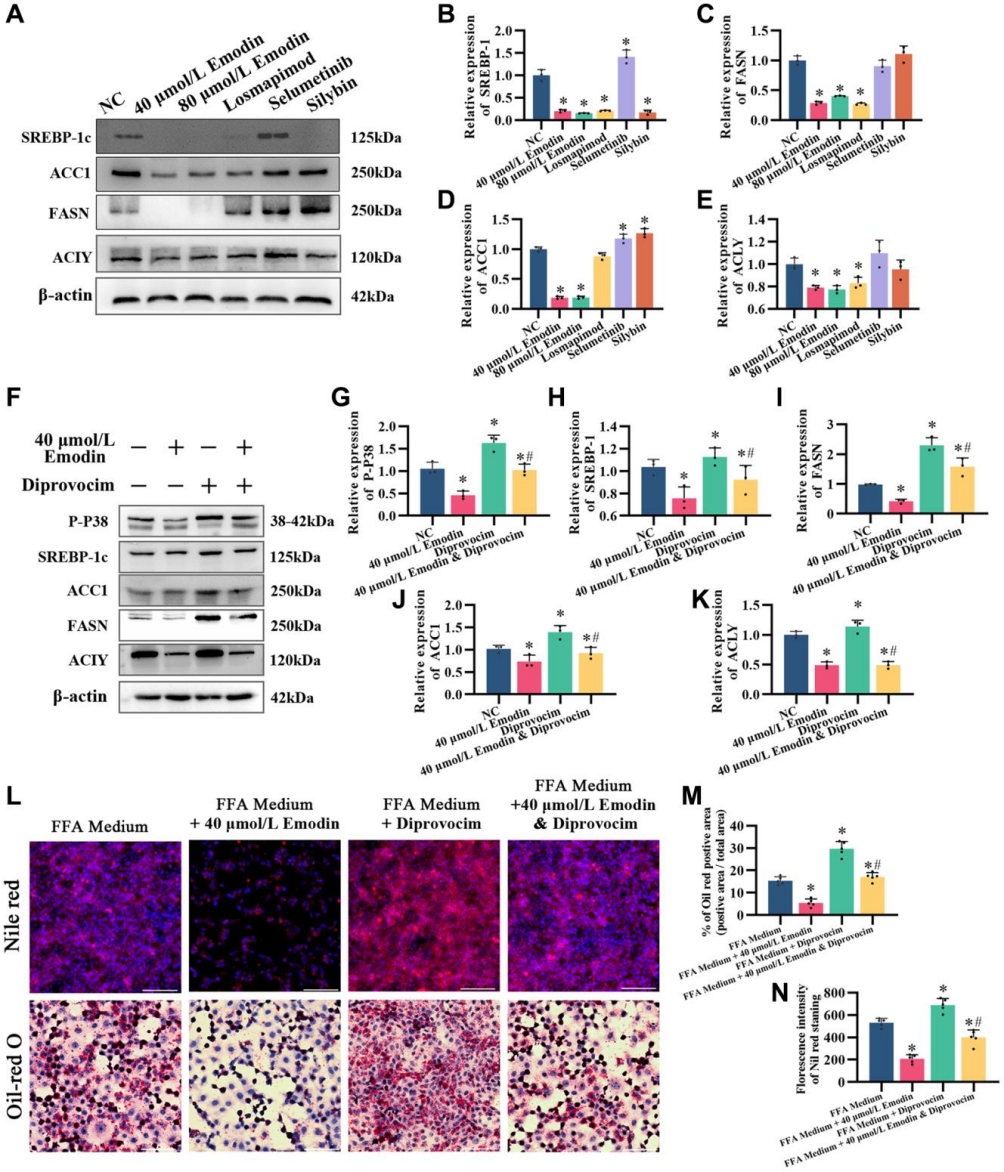
**The MAPK activator diprovocim restored the inhibitory effect of emodin on the AMPK signaling pathway.** (**A**) Representative Western blot of SREBP-1, ACC1, FASN, and ACLY. (**B**–**E**) Western blot analysis. (**F**) Representative Western blot of SREBP-1, ACC1, FASN, ACLY, and P-P38. (**G**–**K**) Western blot analysis. (**L**–**N**) Nile red and Oil red O staining and analysis. ^*^indicates comparison with NC, *P* < 0.05. A indicates a comparison with FFA Medium, *P* < 0.05. The white line indicates 50 μm.

After treatment with Losmapimod, an inhibitor of P38, the expression levels of DNL, including SREBP-1c, ACLY, ACC1, and FASN, were downregulated, similar to emodin intervention (Figure 8A–8E). However, treatments with selumetinib, the MEK inhibitor, and silybin did not yield the same results. Based on this, we speculate that the inhibitory effect of emodin on the AMPK signaling pathway is related to the inhibition of the MAPK p38 signaling pathway.

We utilized diprovocim, a small molecule compound that activates the MAPK signaling pathway, to treat HepG2 cells either alone or in combination with emodin (Figure 8F–8N). The results showed that diprovocim alone promoted the phosphorylation of P38 and upregulated the expression of SREBP-1 in the AMPK signaling pathway, as well as the downstream targets FASN, ACC1, and ACLY. Additionally, simultaneous treatment with diprovocim and emodin restored the inhibitory effect of emodin on SREBP-1, FASN, ACC1, and ACLY (Figure 8F–8K). Moreover, Oil red O staining and Nile red staining showed that simultaneous treatment with both Diprovocim and Emodin increased the accumulation of lipids compared to emodin treatment alone (Figure 8L–8N). These findings suggest that the inhibitory effect of emodin on the AMPK signaling pathway is associated with the inhibition of the MAPK p38 signaling pathway.

## DISCUSSION

The prevalence of NAFLD, which consists of a series of conditions strongly associated with obesity and type 2 diabetes, has climbed to almost a quarter of the world’s population. As a result of its exceptionally complex pathogenesis, approximately 150 monotherapies targeting one or a few genes in clinical trials have not yet been completed or have failed to meet the clinical endpoints, and there are still no monotherapies that have been approved by the Food and Drug Administration [35]. Db/db mice are ideal models of nonalcoholic fatty liver disease [36–38]; db/db mice are often used to build type 2 diabetes models due to the loss of leptin receptor, which leads to weight obesity and increased appetite, while nonalcoholic fatty liver disease is highly related to obesity and type 2 diabetes. Different from the nonalcoholic fatty liver disease induced by high-fat and high-sugar diet, high-fructose diet and choline deficiency diet, the model of db/db mice is stable, and the pure mice will develop into fatty liver over time, to avoid the low rate of diet-induced model, and it is easy to spontaneously alleviate the interference caused by uncontrollable factors such as fatty liver. Therefore, in this study, db/db mice were selected to establish a model of NAFLD. Studies have proven that multiple pathways and interactions affect the pathogenesis of NAFLD [1, 2, 4], including *de novo* lipogenesis enhancement, over esterification of fatty acids, decreases in intrahepatic lipolysis and mitochondrial β-oxidation, and inflammation due to the hepatotoxicity caused by triglyceride overaccumulation in the liver. Each homeostatic dysfunction promotes NAFLD progression and an adverse outcome. Thus, we believe that treatments targeting multiple pathways might achieve better clinical outcomes.

Unlike monotherapy, traditional Chinese medicine focuses on the holistic view of maintaining a homeostatic balance within the whole body; diverse ingredients in traditional Chinese medicines can target a variety of biological factors and regulate multiple signaling pathways. According to how they were prepared, the traditional Chinese medicine herb RPM is classified as Sheng Shou Wu, and PRPM is classified as Zhi Shou Wu [39]. These two different types of RPMs have distinct functions; raw RPMs have high hepatotoxicity, but the toxicity is reduced after processing. After various processing methods, the content of anthraquinones, the main hepatotoxic component of RPM, decreased significantly, which may be the main reason for the reduction in the toxicity of PRPM [40, 41]. Among all of these candidate ingredients, some studies reported a risk of liver injury associated with anthraquinone-related compounds, although systematic studies on the hepatotoxicity of PRPM and its ingredients showed that it does not lead to liver damage if it is administered at a low dose. These studies might explain why clinical studies have reported different or even opposite results on PRPM treatments; diverse dosages or preparation methods of PRPM would cause variations in the PRPM ingredient concentrations, and low-dose PRPM therapy would not significantly impair the liver and even play an active role against NAFLD. Two recent studies have shown that stilbene glycoside, one of the main components of RPM, can hinder emodin metabolism in the liver, expose emodin for a long time, affect bile acid metabolism, and promote emodin to cause obvious hepatotoxicity [42, 43]. The use of emodin or stilbene glycoside alone had no significant effect [44]. It was reported that after RPM was processed, the content of emodin increased and the content of stilbene glycosides decreased, while stilbene glycosides were unstable under acidic conditions and easily degraded into stilbene aglycone at room temperature and further decomposed into phenols [45].

Network pharmacology is a new method based on the theory of systems biology, which analyzes the network of biological systems and selects specific signal nodes for the molecular design of multitarget drugs. Network pharmacology emphasizes the regulation of signaling pathways to improve the therapeutic effect and reduce toxicity and side effects to improve the success rate of new clinical trials and shrink the costs of research and development. Based on network pharmacology, we found that PRPM might have positive effects on NAFLD, and its beneficial mechanisms of action might involve multiple signaling pathways, such as the HIF-1 signaling pathway, AMPK signaling pathway, AGE-RAGE signaling pathway associated with diabetic complications, insulin resistance, and MAPK signaling pathway. Both the AMPK and MAPK signaling pathways play important roles in liver energy metabolism [27, 46]. As a regulator of metabolism and mitochondrial homeostasis, AMPK enhances fatty acid oxidation and lipolysis while inhibiting lipogenesis and glycogen, and therapies targeting AMPK and its downstream targets have been a hot topic in recent years. Additionally, MAPK is a serine/threonine protein kinase that exists widely in mammalian cells. It can be phosphorylated and activated by a series of extracellular stimuli and then conveys extracellular signals to cells and their nuclei, resulting in biological effects. This transduction is unique to eukaryotes and mainly regulates cell growth, proliferation, differentiation, apoptosis and other processes.

MAPK signaling pathway dysfunction was involved in NAFLD; Vernia S. et al. found that liver-specific deletion of Jnk1 and Jnk2 in mice protected mice from diet-induced obesity and insulin resistance [47], while Jing. et al. and Pereira, S. et al. found that p38 α knockout or inhibitor intervention could significantly alleviate diet-induced fatty liver degeneration. In contrast, Khan AS. et al. found that ERK1 whole-body knockout led to increased weight gain as well as increased insulin resistance in diet-induced outcomes, and Kujiraoka, T. et al. found that ERK2 liver tissue-specific knockout led to more adverse liver damage [48]. Therefore, targeted treatment of liver MAPK may be beneficial to NAFLD. In this study, we also found MAPK signaling pathway disruption in the liver. In animal experiments, the expression of the MAPK-related gene P38 was increased in the NAFLD group, and the expression of ERK1/2 was decreased, which is consistent with the results of some clinical studies in nonalcoholic fatty liver groups [49, 50]. Moreover, in the NAFLD groups, the phosphorylation of P38 decreased, while the phosphorylation of ERK1 and ERK2 increased. After treatment for 14 weeks, both high and low doses of PRPM could not only decrease lipid accumulation in the liver but also reverse these changes to varying degrees, suggesting that in addition to DNL-related genes, PRPM therapy may also target MAPK. While we used emodin to interfere with HepG2 cells, we found that emodin can also inhibit the expression of P38 and promote P38 phosphorylation, which is consistent with the findings of PRPM in db/db mice. However, emodin can also inhibit the expression of ERK1/2, while PRPM can upregulate the expression of ERK1/2, indicating that although emodin is one of the active compounds of PRPM, traditional Chinese medicine still interferes with NAFLD in a multicomponent and multitarget way.

In general, through network pharmacology and *in vivo* and *in vitro* experiments, it was found that intervention with PRPM and its main compound emodin could improve lipid droplet accumulation in NAFLD model mice and a fatty liver cell model, and its mechanism may be related to the inhibition of p38/phosphorylated p38. This study provides an experimental foundation for elucidating the mechanism by which PRPM and emodin improve NAFLD.

## Supplementary Materials

Supplementary Materials and Methods

Supplementary Figure 1

Supplementary Tables

## References

[1] Loomba R, Friedman SL, Shulman GI. Mechanisms and disease consequences of nonalcoholic fatty liver disease. Cell. 2021; 184:2537–64. 10.1016/j.cell.2021.04.015 33989548 PMC12168897

[2] Powell EE, Wong VW, Rinella M. Non-alcoholic fatty liver disease. Lancet. 2021; 397:2212–24. 10.1016/S0140-6736(20)32511-3 33894145

[3] Nassir F. NAFLD: Mechanisms, Treatments, and Biomarkers. Biomolecules. 2022; 12:824. 10.3390/biom12060824 35740949 PMC9221336

[4] Dowman JK, Tomlinson JW, Newsome PN. Pathogenesis of non-alcoholic fatty liver disease. QJM. 2010; 103:71–83. 10.1093/qjmed/hcp158 19914930 PMC2810391

[5] Buzzetti E, Pinzani M, Tsochatzis EA. The multiple-hit pathogenesis of non-alcoholic fatty liver disease (NAFLD). Metabolism. 2016; 65:1038–48. 10.1016/j.metabol.2015.12.012 26823198

[6] Ress C, Kaser S. Mechanisms of intrahepatic triglyceride accumulation. World J Gastroenterol. 2016; 22:1664–73. 10.3748/wjg.v22.i4.1664 26819531 PMC4721997

[7] Xu Y, Li H, Chen G, Zhu L, Lin H, Huang C, Wei S, Yang T, Qian W, Li X, Zhao S, Pan W. Radix polygoni multiflori protects against hippocampal neuronal apoptosis in diabetic encephalopathy by inhibiting the HDAC4/JNK pathway. Biomed Pharmacother. 2022; 153:113427. 10.1016/j.biopha.2022.113427 36076547

[8] Lin HQ, Ho MT, Lau LS, Wong KK, Shaw PC, Wan DC. Anti-acetylcholinesterase activities of traditional Chinese medicine for treating Alzheimer's disease. Chem Biol Interact. 2008; 175:352–4. 10.1016/j.cbi.2008.05.030 18573242

[9] Ning F, Chen L, Chen L, Liu X, Zhu Y, Hu J, Xie G, Xia J, Shi K, Lan Z, Wang P. Combination of Polygoni Multiflori Radix Praeparata and Acori Tatarinowii Rhizoma Alleviates Learning and Memory Impairment in Scopolamine-Treated Mice by Regulating Synaptic-Related Proteins. Front Pharmacol. 2021; 12:679573. 10.3389/fphar.2021.679573 34393775 PMC8360279

[10] Lin EY, Bayarsengee U, Wang CC, Chiang YH, Cheng CW. The natural compound 2,3,5,4'-tetrahydroxystilbene-2-O-β-d glucoside protects against adriamycin-induced nephropathy through activating the Nrf2-Keap1 antioxidant pathway. Environ Toxicol. 2018; 33:72–82. 10.1002/tox.22496 29064158

[11] Liu X, Yang C, Deng Y, Liu P, Yang H, Du X, Du Y. Polygoni Multiflori Radix Preparat Delays Skin Aging by Inducing Mitophagy. Biomed Res Int. 2021; 2021:5847153. 10.1155/2021/5847153 33511202 PMC7822667

[12] He Y, Wang F, Chen S, Liu M, Pan W, Li X. The Protective Effect of Radix Polygoni Multiflori on Diabetic Encephalopathy via Regulating Myosin Light Chain Kinase Expression. J Diabetes Res. 2015; 2015:484721. 10.1155/2015/484721 26199947 PMC4496489

[13] Li H, Wang X, Liu Y, Pan D, Wang Y, Yang N, Xiang L, Cai X, Feng Y. Hepatoprotection and hepatotoxicity of Heshouwu, a Chinese medicinal herb: Context of the paradoxical effect. Food Chem Toxicol. 2017; 108:407–18. 10.1016/j.fct.2016.07.035 27484243

[14] Wu F, Li Y, Liu W, Xiao R, Yao B, Gao M, Xu D, Wang J. Comparative Investigation of Raw and Processed Radix Polygoni Multiflori on the Treatment of Vascular Dementia by Liquid Chromatograph-Mass Spectrometry Based Metabolomic Approach. Metabolites. 2022; 12:1297. 10.3390/metabo12121297 36557335 PMC9785642

[15] Huang R, Li R, Chen J, Lv M, Xu X. Network pharmacology analysis of the pharmacological mechanism of Artemisia lavandulaefolia DC. in rheumatoid arthritis. Phytomedicine. 2023; 118:154905. 10.1016/j.phymed.2023.154905 37348247

[16] Qu J, Ke F, Liu Z, Yang X, Li X, Xu H, Li Q, Bi K. Uncovering the mechanisms of dandelion against triple-negative breast cancer using a combined network pharmacology, molecular pharmacology and metabolomics approach. Phytomedicine. 2022; 99:153986. 10.1016/j.phymed.2022.153986 35183931

[17] Moodley D, Yoshida H, Mostafavi S, Asinovski N, Ortiz-Lopez A, Symanowicz P, Telliez JB, Hegen M, Clark JD, Mathis D, Benoist C. Network pharmacology of JAK inhibitors. Proc Natl Acad Sci U S A. 2016; 113:9852–7. 10.1073/pnas.1610253113 27516546 PMC5024632

[18] Yang JB, Song YF, Liu Y, Gao HY, Wang Q, Wang Y, Cheng XL, Zuo TT, Hu XW, Wei F, Jin HT, Wang ST, Ma SC. UHPLC-QQQ-MS/MS assay for the quantification of dianthrones as potential toxic markers of Polygonum multiflorum Thunb: applications for the standardization of traditional Chinese medicines (TCMs) with endogenous toxicity. Chin Med. 2021; 16:51. 10.1186/s13020-021-00463-w 34217329 PMC8254911

[19] Luo DQ, Jia P, Zhao SS, Zhao Y, Liu HJ, Wei F, Ma SC. Identification and Differentiation of Polygonum multiflorum Radix and Polygoni multiflori Radix Preaparata through the Quantitative Analysis of Multicomponents by the Single-Marker Method. J Anal Methods Chem. 2019; 2019:7430717. 10.1155/2019/7430717 31485368 PMC6702820

[20] Liang L, Xu J, Zhou WW, Brand E, Chen HB, Zhao ZZ. Integrating Targeted and Untargeted Metabolomics to Investigate the Processing Chemistry of Polygoni Multiflori Radix. Front Pharmacol. 2018; 9:934. 10.3389/fphar.2018.00934 30210339 PMC6121093

[21] Wang S, Sun X, An S, Sang F, Zhao Y, Yu Z. High-Throughput Identification of Organic Compounds from Polygoni Multiflori Radix Praeparata (*Zhiheshouwu*) by UHPLC-Q-Exactive Orbitrap-MS. Molecules. 2021; 26:3977. 10.3390/molecules26133977 34209934 PMC8428211

[22] Chen SC, Yang CS, Chen JJ. Main Bioactive Components and Their Biological Activities from Natural and Processed Rhizomes of Polygonum sibiricum. Antioxidants (Basel). 2022; 11:1383. 10.3390/antiox11071383 35883874 PMC9311596

[23] Carriel V, Campos A, Alaminos M, Raimondo S, Geuna S. Staining Methods for Normal and Regenerative Myelin in the Nervous System. Methods Mol Biol. 2017; 1560:207–18. 10.1007/978-1-4939-6788-9_15 28155156

[24] Donohoe F, Wilkinson M, Baxter E, Brennan DJ. Mitogen-Activated Protein Kinase (MAPK) and Obesity-Related Cancer. Int J Mol Sci. 2020; 21:1241. 10.3390/ijms21041241 32069845 PMC7072904

[25] Zhang X, Fan L, Wu J, Xu H, Leung WY, Fu K, Wu J, Liu K, Man K, Yang X, Han J, Ren J, Yu J. Macrophage p38α promotes nutritional steatohepatitis through M1 polarization. J Hepatol. 2019; 71:163–74. 10.1016/j.jhep.2019.03.014 30914267

[26] Lan T, Hu Y, Hu F, Li H, Chen Y, Zhang J, Yu Y, Jiang S, Weng Q, Tian S, Ma T, Yang G, Luo D, et al. Hepatocyte glutathione S-transferase mu 2 prevents non-alcoholic steatohepatitis by suppressing ASK1 signaling. J Hepatol. 2022; 76:407–19. 10.1016/j.jhep.2021.09.040 34656650

[27] Lawan A, Bennett AM. Mitogen-Activated Protein Kinase Regulation in Hepatic Metabolism. Trends Endocrinol Metab. 2017; 28:868–78. 10.1016/j.tem.2017.10.007 29128158 PMC5774993

[28] Salloum S, Jeyarajan AJ, Kruger AJ, Holmes JA, Shao T, Sojoodi M, Kim MH, Zhuo Z, Shroff SG, Kassa A, Corey KE, Khan SK, Lin W, et al. Fatty Acids Activate the Transcriptional Coactivator YAP1 to Promote Liver Fibrosis via p38 Mitogen-Activated Protein Kinase. Cell Mol Gastroenterol Hepatol. 2021; 12:1297–310. 10.1016/j.jcmgh.2021.06.003 34118488 PMC8463869

[29] Yuan J, Dong X, Yap J, Hu J. The MAPK and AMPK signalings: interplay and implication in targeted cancer therapy. J Hematol Oncol. 2020; 13:113. 10.1186/s13045-020-00949-4 32807225 PMC7433213

[30] Win S, Min RWM, Zhang J, Kanel G, Wanken B, Chen Y, Li M, Wang Y, Suzuki A, Aung FWM, Murray SF, Aghajan M, Than TA, Kaplowitz N. Hepatic Mitochondrial SAB Deletion or Knockdown Alleviates Diet-Induced Metabolic Syndrome, Steatohepatitis, and Hepatic Fibrosis. Hepatology. 2021; 74:3127–45. 10.1002/hep.32083 34331779 PMC8639630

[31] Kheiripour N, Karimi J, Khodadadi I, Tavilani H, Taghi Goodarzi M, Hashemnia M. Hepatoprotective Effects of Silymarin on Liver Injury via Irisin Upregulation and Oxidative Stress Reduction in Rats with Type 2 Diabetes. Iran J Med Sci. 2019; 44:108–17. 30936597 PMC6423431

[32] Lee HA, Chang Y, Sung PS, Yoon EL, Lee HW, Yoo JJ, Lee YS, An J, Song DS, Cho YY, Kim SU, Kim YJ. Therapeutic mechanisms and beneficial effects of non-antidiabetic drugs in chronic liver diseases. Clin Mol Hepatol. 2022; 28:425–72. 10.3350/cmh.2022.0186 35850495 PMC9293616

[33] Ren L, Ma XL, Wang HL, Li R, Cui JJ, Yan PJ, Wang YN, Yu XY, Du P, Yu HY, Guo HH, Tang R, Che YS, et al. Prebiotic-like cyclodextrin assisted silybin on NAFLD through restoring liver and gut homeostasis. J Control Release. 2022; 348:825–40. 10.1016/j.jconrel.2022.06.031 35752255

[34] Yu L, Gong L, Wang C, Hu N, Tang Y, Zheng L, Dai X, Li Y. Radix Polygoni Multiflori and Its Main Component Emodin Attenuate Non-Alcoholic Fatty Liver Disease in Zebrafish by Regulation of AMPK Signaling Pathway. Drug Des Devel Ther. 2020; 14:1493–506. 10.2147/DDDT.S243893 32346285 PMC7167271

[35] Ferguson D, Finck BN. Emerging therapeutic approaches for the treatment of NAFLD and type 2 diabetes mellitus. Nat Rev Endocrinol. 2021; 17:484–95. 10.1038/s41574-021-00507-z 34131333 PMC8570106

[36] Feng J, Li L, Ou Z, Li Q, Gong B, Zhao Z, Qi W, Zhou T, Zhong J, Cai W, Yang X, Zhao A, Gao G, Yang Z. IL-25 stimulates M2 macrophage polarization and thereby promotes mitochondrial respiratory capacity and lipolysis in adipose tissues against obesity. Cell Mol Immunol. 2018; 15:493–505. 10.1038/cmi.2016.71 28194019 PMC6068125

[37] Park M, Sharma A, Baek H, Han JY, Yu J, Lee HJ. Stevia and Stevioside Attenuate Liver Steatosis through PPARα-Mediated Lipophagy in db/db Mice Hepatocytes. Antioxidants (Basel). 2022; 11:2496. 10.3390/antiox11122496 36552704 PMC9774531

[38] Suriano F, Vieira-Silva S, Falony G, Roumain M, Paquot A, Pelicaen R, Régnier M, Delzenne NM, Raes J, Muccioli GG, Van Hul M, Cani PD. Novel insights into the genetically obese (ob/ob) and diabetic (db/db) mice: two sides of the same coin. Microbiome. 2021; 9:147. 10.1186/s40168-021-01097-8 34183063 PMC8240277

[39] Huang J, Zhang JP, Bai JQ, Wei MJ, Zhang J, Huang ZH, Qu GH, Xu W, Qiu XH. Chemical profiles and metabolite study of raw and processed Polygoni Multiflori Radix in rats by UPLC-LTQ-Orbitrap MS(n) spectrometry. Chin J Nat Med. 2018; 16:375–400. 10.1016/S1875-5364(18)30070-0 29860999

[40] Du KZ, Chen Y, Li J, Tang G, Tian F, He J, Chang Y. Quantification of eight active ingredients in crude and processed radix polygoni multiflori applying miniaturized matrix solid-phase dispersion microextraction followed by UHPLC. J Sep Sci. 2018; 41:3486–95. 10.1002/jssc.201800342 30028075

[41] Liang Z, Chen H, Yu Z, Zhao Z. Comparison of raw and processed Radix Polygoni Multiflori (Heshouwu) by high performance liquid chromatography and mass spectrometry. Chin Med. 2010; 5:29. 10.1186/1749-8546-5-29 20704710 PMC2930642

[42] Ma J, Zheng L, Deng T, Li CL, He YS, Li HJ, Li P. Stilbene glucoside inhibits the glucuronidation of emodin in rats through the down-regulation of UDP-glucuronosyltransferases 1A8: application to a drug-drug interaction study in Radix Polygoni Multiflori. J Ethnopharmacol. 2013; 147:335–40. 10.1016/j.jep.2013.03.013 23523942

[43] Yu Q, Jiang LL, Luo N, Fan YX, Ma J, Li P, Li HJ. Enhanced absorption and inhibited metabolism of emodin by 2, 3, 5, 4'-tetrahydroxystilbene-2-O-β-D-glucopyranoside: Possible mechanisms for Polygoni Multiflori Radix-induced liver injury. Chin J Nat Med. 2017; 15:451–7. 10.1016/S1875-5364(17)30067-5 28629535

[44] Tu C, Gao D, Li XF, Li CY, Li RS, Zhao YL, Li N, Jia GL, Pang JY, Cui HR, Ma ZJ, Xiao XH, Wang JB. Corrigendum: Inflammatory Stress Potentiates Emodin-Induced Liver Injury in Rats. Front Pharmacol. 2021; 11:597772. 10.3389/fphar.2020.597772 33568998 PMC7869054

[45] Zhao MJ, Gong XH, Dang J, Yuan A, Li Y, Lou L, Liu MC, Li YX. [Effect of processing time of Polygoni Multiflori Radix on content changes of 16 componens]. Zhongguo Zhong Yao Za Zhi. 2017; 42:1344–9. 10.19540/j.cnki.cjcmm.20170121.030 29052397

[46] Day EA, Ford RJ, Steinberg GR. AMPK as a Therapeutic Target for Treating Metabolic Diseases. Trends Endocrinol Metab. 2017; 28:545–60. 10.1016/j.tem.2017.05.004 28647324

[47] Vernia S, Cavanagh-Kyros J, Garcia-Haro L, Sabio G, Barrett T, Jung DY, Kim JK, Xu J, Shulha HP, Garber M, Gao G, Davis RJ. The PPARα-FGF21 hormone axis contributes to metabolic regulation by the hepatic JNK signaling pathway. Cell Metab. 2014; 20:512–25. 10.1016/j.cmet.2014.06.010 25043817 PMC4156535

[48] Kujiraoka T, Satoh Y, Ayaori M, Shiraishi Y, Arai-Nakaya Y, Hakuno D, Yada H, Kuwada N, Endo S, Isoda K, Adachi T. Hepatic extracellular signal-regulated kinase 2 suppresses endoplasmic reticulum stress and protects from oxidative stress and endothelial dysfunction. J Am Heart Assoc. 2013; 2:e000361. 10.1161/JAHA.113.000361 23954796 PMC3828781

[49] González-Terán B, Matesanz N, Nikolic I, Verdugo MA, Sreeramkumar V, Hernández-Cosido L, Mora A, Crainiciuc G, Sáiz ML, Bernardo E, Leiva-Vega L, Rodríguez E, Bondía V, et al. p38γ and p38δ reprogram liver metabolism by modulating neutrophil infiltration. EMBO J. 2016; 35:536–52. 10.15252/embj.201591857 26843485 PMC4772851

[50] Meijnikman AS, van Olden CC, Aydin Ö, Herrema H, Kaminska D, Lappa D, Männistö V, Tremaroli V, Olofsson LE, de Brauw M, van de Laar A, Verheij J, Gerdes VEA, et al. Hyperinsulinemia Is Highly Associated With Markers of Hepatocytic Senescence in Two Independent Cohorts. Diabetes. 2022; 71:1929–36. 10.2337/db21-1076 35713877 PMC9450852

