# Supplementary material for: Prepared Radix Polygoni Multiflori and emodin alleviate lipid droplet accumulation in nonalcoholic fatty liver disease through MAPK signaling pathway inhibition
Source: Aging (Albany NY). 2024 Jan 26;16(3):2362–84. doi: 10.18632/aging.205485 (PMC10911387; doi:10.18632/aging.205485)
Supplement: Supplementary Materials and Methods [file aging-16-205485-s001.pdf]

SUPPLEMENTARY MATERIALS AND METHODS

1 Preanalytical sample handling UPLC–MS/MS mass spectrometry and liquid phase methods

Sample pretreatment: Mice were euthanized 15 min after gavage, and liver tissue samples were obtained according to previous studies and references [1, 2]. Then, 0.4 ml of saline was added to the tissue homogenate, methanol at triple volume was added for precipitating the protein, and the turbine was mixed for 10 s, filtering supernatants via 0.22 µm filters head for instrumental analysis. The components of the PRPM extract were identified by using Waters ACQUITY UPLC I-Class (Waters, Milford, MA, USA) and Xevo TQD IVD (Waters, Milford, MA, USA) instruments. Preparation of controls: Appropriate amounts of emodin (CAS: 518-82-1, Beijing Solarbio Science and Technology Co., Ltd., China), tetrahydroxystilbene glucoside (CAS: 873-94-2, Beijing Solarbio Science and Technology Co., Ltd., China), and physcion (CAS: 521-61-9, Beijing Solarbio Science and Technology Co., Ltd.) were weighed. Standards were placed in a 10 ml measuring flask, dissolved in methanol and then diluted precisely to the needed concentration of the control solution when used. Mass spectrometry conditions: ionization at an electrospray ionization source (ESI), multiple reaction monitoring (MRM) detection in positive ion mode, Capillay (kV): 2.50, Desolvation Temp (°C): 200. The MRM of emodin, tetrahydroxystilbene glucoside and physcion are shown in Supplementary Table 1. Chromatographic conditions. The separation was carried out on an ACQUITY UPLC BEH C18 (2.1 × 50 mm, 1.7 µm) column using the mobile phase: (A) 0.1% formic acid water-acetonitrile and (B) binary gradient elution; flow rate: 0.2 ml/min. Column temperature 35°C, injection volume 1 µl. Gradient elution program: 0–1 min (30% A), 1–2 min (30%-90% A), 2–3 min (90% A), 3-4 min (90–30% A), 4–5 min (30% A).

RESULTS

1 UPLC–MS/MS analysis of the prepared radix polygoni multiflori active compounds in the mouse liver

We reviewed the conversion measurements provided by the Chinese Pharmacopoeia and previous studies and divided PRPM treatment into a high-dose group and a low-dose group to investigate whether different preparations and doses of PRPM play different roles in the liver. In this study, the PRPM active compounds gathered in hepatic tissues after gavage were extracted after 40–50% ethanol precipitation for 48 hours, and UPLC–MS/MS tests were performed to detect the components of emodin, tetrahydroxystilbene glucoside, and physcion (Supplementary Figure 1B) (The mass spectrometry conditions are shown in Supplementary Table 2). The results showed that we detected the peaks of the three active compounds at retention times of 0.32 s, 0.78 s and 3.27 s (Supplementary Figure 1A). A comparison of network pharmacological analysis and relevant literature references revealed that emodin may be the main active ingredient in PRPM that is responsible for the improvement of nonalcoholic fatty liver disease.

SUPPLEMENTARY REFERENCES

1. Rainville PD, Simeone JL, McCarthy SM, Smith NW, Cowan D, Plumb RS. Investigation of microbore UPLC and nontraditional mobile phase compositions for bioanalytical LC-MS/MS. Bioanalysis. 2012; 4:1287–97. doi: 10.4155/bio.12.78. PMID: 22720648. https://doi.org/10.4155/bio.12.78 PMID:22720648

2. Song Y, Yang J, Wang X, Chen J, Si D, Gao H, Sun M, Cheng X, Wei F, Ma S. Pharmacokinetics and metabolism of trans-emodin dianthrones in rats. J Ethnopharmacol. 2022; 290:115123. https://doi.org/10.1016/j.jep.2022.115123 PMID:35183691
