# Supplementary material for: Prepared Radix Polygoni Multiflori and emodin alleviate lipid droplet accumulation in nonalcoholic fatty liver disease through MAPK signaling pathway inhibition
Source: Aging (Albany NY). 2024 Jan 26;16(3):2362–84. doi: 10.18632/aging.205485 (PMC10911387; doi:10.18632/aging.205485)
Supplement: Supplementary Figure 1 [file aging-16-205485-s002.pdf]

## SUPPLEMENTARY FIGURE

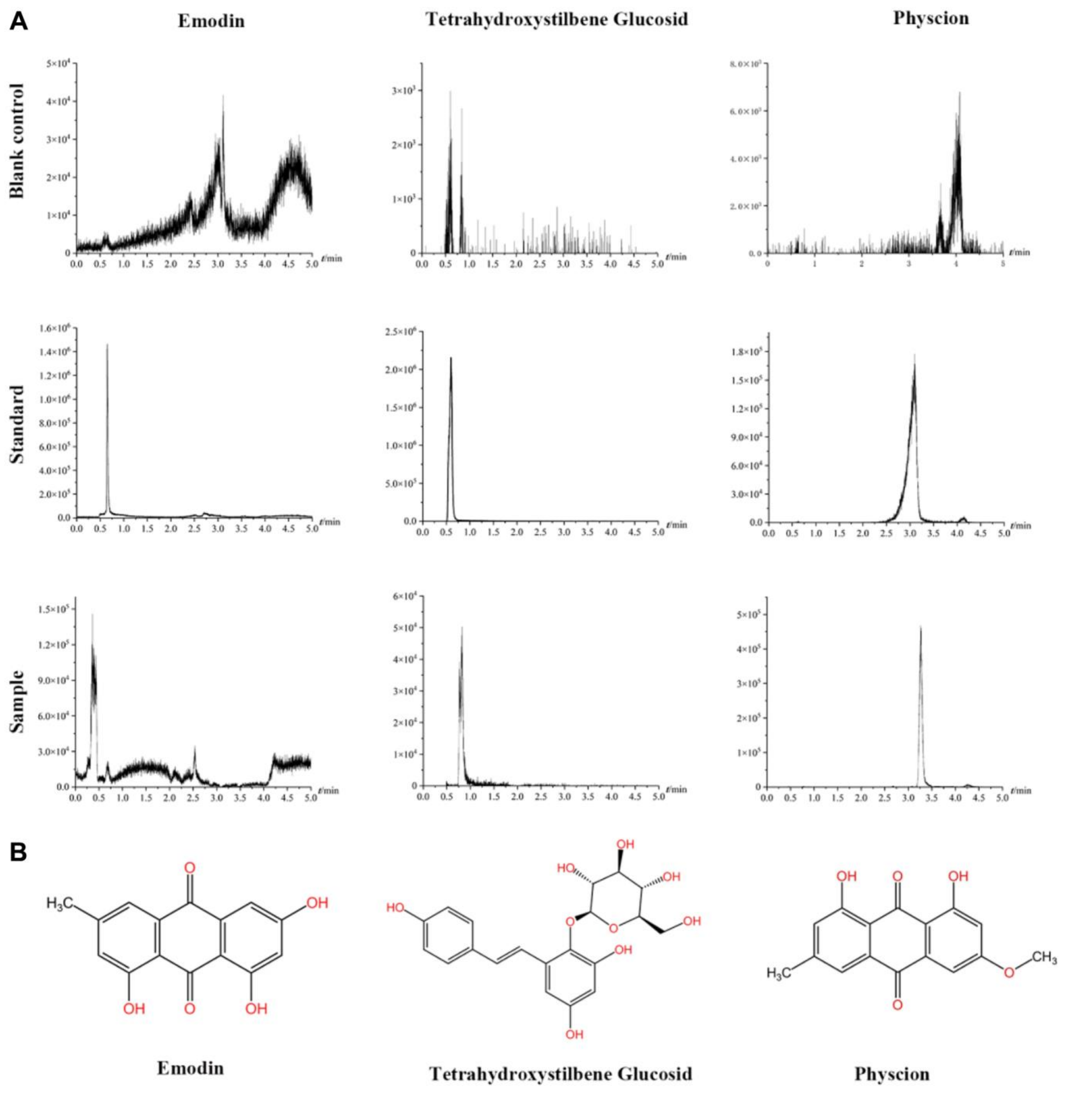


**Supplementary Figure 1. The three active ingredients of PRPM in mouse liver as measured by UPLC–MS/MS.** (**A**) Chromatograms of three active components. (**B**) Chemical structure of the three active ingredients.
