# Supplementary material for: Prepared Radix Polygoni Multiflori and emodin alleviate lipid droplet accumulation in nonalcoholic fatty liver disease through MAPK signaling pathway inhibition
Source: Aging (Albany NY). 2024 Jan 26;16(3):2362–84. doi: 10.18632/aging.205485 (PMC10911387; doi:10.18632/aging.205485)
Supplement: Supplementary Tables [file aging-16-205485-s003.pdf]

SUPPLEMENTARY TABLES

Supplementary Table 1. OB and DL scores of each PRPM ingredients.

Ingredient	OB (%)	DL
n-trans-feruloyltyramine	86.71	0.26
aloeemodin	83.38	0.24
rhein	47.07	0.28
Physciondiglucoside	41.65	0.63
sitosterol	36.91	0.75
procyanidin b-13′-o-gallate	31.99	0.32
gallic acid	31.69	0.04
guaijaverin	29.65	0.70
chrysazin	28.74	0.19
tricin	27.86	0.34
emodinanthrone	24.72	0.21
questinol	24.49	0.30
emodin	24.40	0.24
physcion	22.29	0.27
citreorosein	22.19	0.27
piceid	21.44	0.50
daucosterol	20.63	0.63
questin	20.44	0.27
resveratrol	19.07	0.11
6-methoxyluteolin-7-glucoside	19.00	0.81
chrysophanic	18.64	0.21
chrysophanol	18.64	0.21
physcion-8-o-beta-d-glucoside	18.31	0.63
emodin-8-o-beta-d-glucoside	10.03	0.80
polygalacic acid	8.95	0.70
physcion 1-o-beta-d-glucoside	8.20	0.85
3,4,3′,5′-Tetrahydroxystilbene-3-glucoside	2.99	0.55
Rhein diglucoside	2.93	0.63
chrysophanol-8-O-beta-D-(6′-O-galloyl)-glucopyranoside	1.92	0.69

Supplementary Table 2. The mass spectrometry conditions.

Composition	Parent ion	Daughter ion	Cone	Collision energy
Emodin	270.84	114.96	50	46
Tetrahydroxystilbene Glucosid	407.03	245.04	26	10
Physcion	284.94	139.11	56	58
